# Supplementary figures and images for: The importance of local settings: within-year variability in seawater temperature at South Bay, Western Antarctic Peninsula
Source: PeerJ. 2018 Jan 18;6:e4289. doi: 10.7717/peerj.4289 (PMC5776021; doi:10.7717/peerj.4289)

# YELCHO STATION Bay\_PY1\_10 m

## Daily data

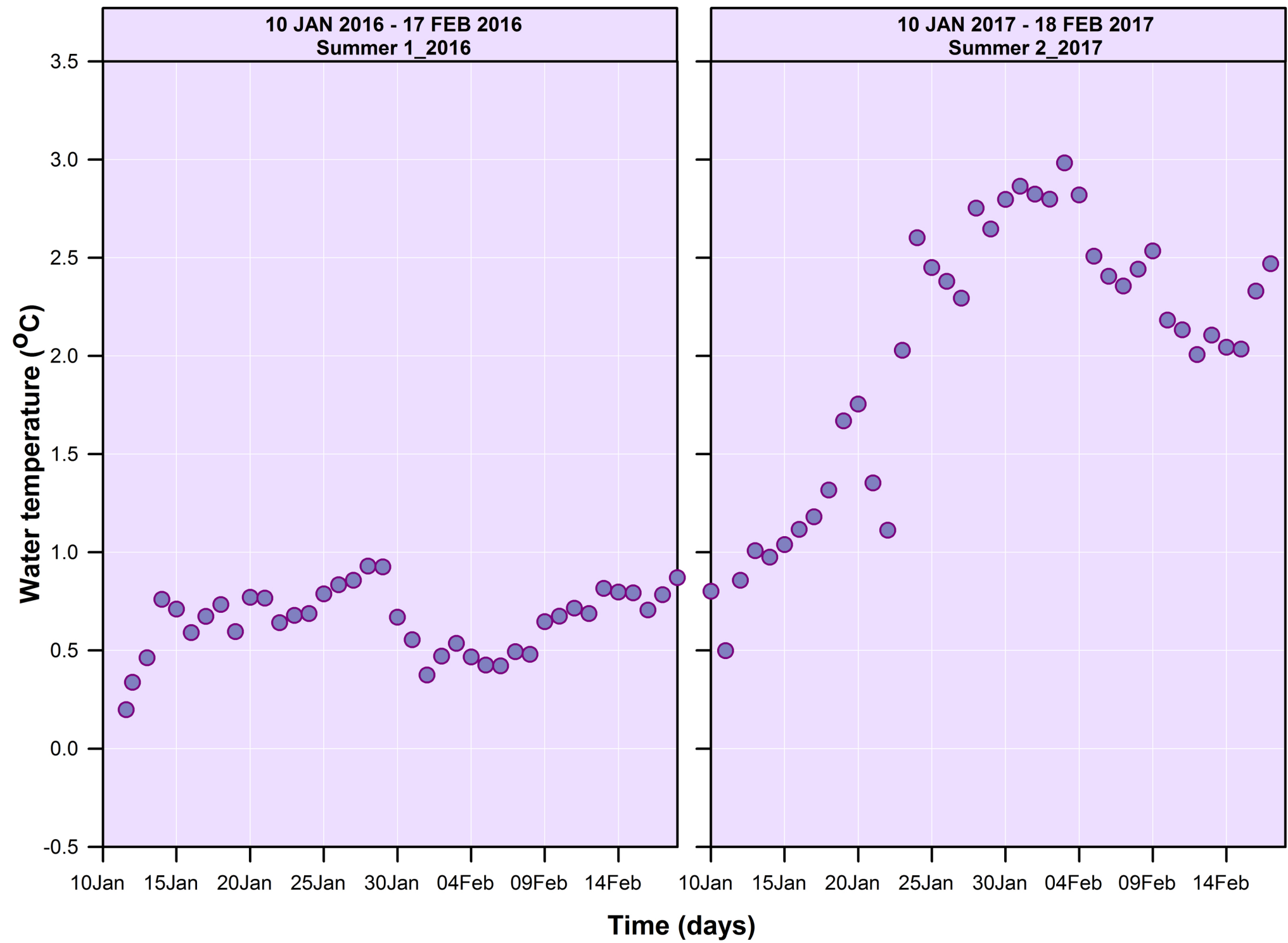

Supplement: Supplemental Information 7 — Seawater temperature recorded at 10 m in summer (January-February) 2016 and 2017 at Doumer Island, WAP. Dots represent daily means. [file peerj-06-4289-s007.pdf]

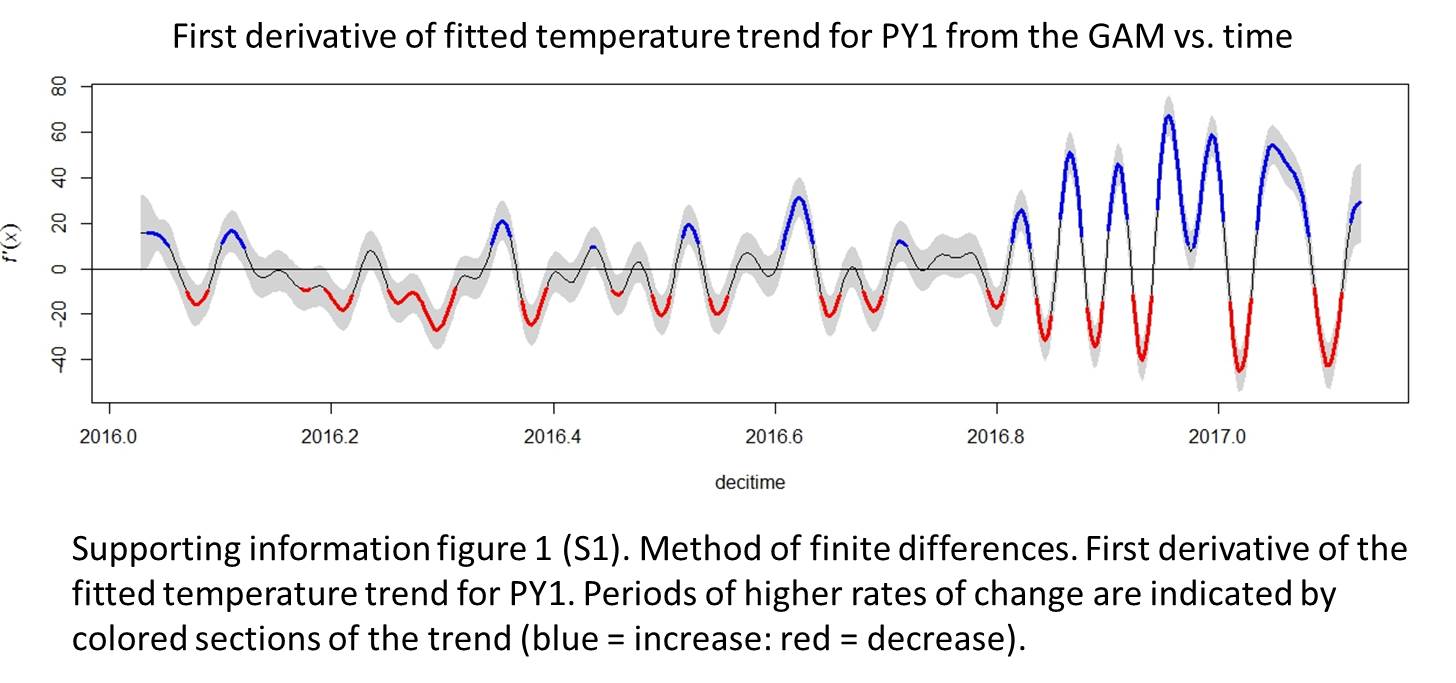

Supplement: Supplemental Information 8 — First derivative of the fitted temperature trend for PY1. Periods of higher rates of change are indicated by coloured sections of the trend. [file peerj-06-4289-s008.jpg]
