# Supplementary material for: The importance of local settings: within-year variability in seawater temperature at South Bay, Western Antarctic Peninsula
Source: PeerJ. 2018 Jan 18;6:e4289. doi: 10.7717/peerj.4289 (PMC5776021; doi:10.7717/peerj.4289)
Supplement: Supplemental Information 9 — Within-year Generalized Additive model (GAM) for PY1, PY2 and PY4. PY1_Method of finite differences (Curtis & Simpson, 2014; Monteith et al., 2014). [file peerj-06-4289-s009.docx]

**R CODE_PeerJ The importance of local natural variability (Cardenas et al.,)**

**Within-year Generalized Additive model (GAM) for PY1**

require(nlme)

require(mgcv)

require(TSA)

require(lubridate)

***##Data***

PY1_Jday <-read.csv(file="EXPORT YELCHO/ peerj-19157-PY1_Jday.csv",stringsAsFactors = FALSE)

class(PY1_Jday)

names(PY1_Jday)

str(PY1_Jday)

PY1_Jday

***###Decimal day date transformation***

PY1_Jday $Datetime <- as.POSIXct(PY1_Jday $Datetime,format = "%m/%d/%Y %H:%M")

PY1_Jday <- transform(PY1_Jday, decitime = decimal_date(Datetime))

str(PY1_Jday)

plot(PY1_Jday $Datetime, PY1_Jday $mean_TEMP, pch=16)

***###:::YP1***

YP1gam1<-gam(mean_TEMP ~ s(decitime),data = PY1_Jday)

summary(YP1gam1)

gam.check(YP1gam1)

par(mfrow=c(1,1))

plot(YP1gam1, scheme=1)

plot(YP1gam1,all.terms = T, shift = -0.33240 , ylab= "Mean Day Water Tempearture (C)", xlab = "Time (days)", ylim=c(min(-1.5),max(3.2)),main="gam1 YELCHOP1 /n seasonality within a year", scheme = 1, pages=1)

points(PY1_Jday$decitime, PY1_Jday$mean_TEMP, pch=16, cex=0.6)

***###___GAM______GAM_checks for autocorrelation***

par(mfrow=c(1,2))

acf(residuals(YP1gam1))

pacf(residuals(YP1gam1))

par(mfrow=c(1,1))

***###___GAM______helps to find ARMA structure***

eacf(residuals(YP1gam1))

***##::::GAMM::::::::::Autoregression structure GAM1 final***

YP1_AR1MA1 <- (gamm(mean_TEMP ~ s(decitime), data = PY1_Jday, correlation = corARMA(p=1, q = 1)))

anova(YP1gamm1$lme,YP1_AR1$lme,YP1_AR1MA1$lme,YP1_AR1MA2$lme)#to choose

plot(YP1_AR1MA1$gam,all.terms = T, shift = -0.31245 , ylab= "Mean Day Water Tempearture (C)", xlab = "Time (days)", ylim=c(min(-1.5),max(3.2)),main="gam1AR1MA2 YELCHOP1 /n seasonality within a year", scheme = 1, pages=1)

points(PY1_Jday$decitime, PY1_Jday$mean_TEMP, pch=16, cex=0.6)

***###FOR GAMM***

summary(YP1_AR1MA1$gam)

layout(matrix(1:1, ncol = 1))

plot(YP1_AR1MA1$gam, scale = 0)

layout(1)

reeeei<-resid(YP1_AR1MA1$lme, type = "normalized")

par(mfrow=c(1,2))

acf(reeeei, lag.max = 300, main = "ACF - ARMA errors/YP1_AR1MA1")

pacf(reeeei, lag.max = 300, main = "pACF- ARMA errors/YP1_AR1MA1")

eacf(reeeei)

acf(reeeei, plot=FALSE)$acf[2]

**PY2**

***###Data***

PY2_Jday <-read.csv(file="EXPORT YELCHO/ peerj-19157-PY2_Jday.csv",stringsAsFactors = FALSE)

class(PY2_Jday)

names(PY2_Jday)

str(PY2_Jday)

PY2_Jday

***###Decimal day date transformation***

PY2_Jday$Datetime20m <- as.POSIXct(PY2_Jday $Datetime20m,format = "%m/%d/%Y %H:%M")

PY2_Jday <- transform(PY2_Jday, decitimeP20 = decimal_date(Datetime20m))

str(PY2_Jday)

par(mfrow=c(1,1))

plot(PY2_Jday$Datetime20m, PY2_Jday$mean_TEMP20m, pch=16)

***###:::YP2***

names (PY2_Jday)

YP20gam1<-gam(mean_TEMP20m ~ s(decitimeP20),data = PY2_Jday)###final

***###___GAM______FOR GAM***

summary(YP20gam1)

***###___GAM______GAM_check residuals***

gam.check(YP20gam1)

***###___GAM______GAM_check effect plot***

par(mfrow=c(1,2))

plot(YP20gam1, scheme=1)

***###___GAM______GAM check response plots***

plot(YP20gam1,all.terms = T, shift = -0.51117 , ylab= "Mean Day Water Tempearture (C)",

xlab = "Time (days)", ylim=c(min(-1.5),max(3.2)),main="gam1 YELCHO P20m /n variability pattern within the period", scheme = 1, pages=1)

points(PY2_Jday $decitimeP20, PY2_Jday $mean_TEMP20m, pch=16, cex=0.6)

***###___GAM______GAM_check for autocorrelation***

par(mfrow=c(1,2))

acf(residuals(YP20gam1))

pacf(residuals(YP20gam1))

***###___GAM______helps to find ARMA structure***

eacf(residuals(YP20gam1))

***###::::GAMM::::::::::Autoregression structure GAM1***

YP20_AR1 <- (gamm(mean_TEMP20m ~ s(decitimeP20), data = PY2_Jday, correlation = corARMA(p=1)))

##anova(YP20gamm1$lme,YP20_AR1$lme,YP20_AR1MA1$lme, YP20_AR1MA2$lme)

names(PY2_Jday)

par(mfrow=c(1,1))

plot(YP20_AR1$gam,all.terms = T, shift = -0.50662 , ylab= "Mean Day Water Tempearture (C)", xlab = "Time (days)", ylim=c(min(-1.5),max(3.2)),main="gam1AR1 YELCHOP4 /n seasonality within a year", scheme = 1, pages=1)

points(PY2_Jday $decitimeP20, PY2_Jday $mean_TEMP20m, pch=16, cex=0.6)

***###FOR GAMM***

summary(YP20_AR1$gam)

layout(matrix(1:1, ncol = 1))

plot(YP20_AR1$gam, scale = 0)

layout(1)

RREESS<-resid(YP20_AR1$lme, type = "normalized")

par(mfrow=c(1,2))

acf(RREESS, lag.max = 300, main = "ACF - ARMA errors/YP20_AR1")

pacf(RREESS, lag.max = 300, main = "pACF- ARMA errors/YP20_AR1")

eacf(RREESS)

acf(RREESS, plot=FALSE)$acf[2]

**PY4**

***###Data***

PY4_Jday <-read.csv(file="EXPORT YELCHO/ peerj-19157-PY4_Jday.csv",stringsAsFactors = FALSE)

class(PY4_Jday)

names(PY4_Jday)

str(PY4_Jday)

PY4_Jday

***###Decimal day date transformation***

PY4_Jday $Datetimep4 <- as.POSIXct(PY4_Jday $Datetimep4,format = "%m/%d/%Y %H:%M")

PY4_Jday <- transform(PY4_Jday, decitimeP4 = decimal_date(Datetimep4))

str(PY4_Jday)

par(mfrow=c(1,1))

plot(PY4_Jday $Datetimep4, PY4_Jday $mean_TEMp4, pch=16)

***###:::YP4_final***

names (PY4_Jday)

YP4gam1<-gam(mean_TEMp4 ~ s(decitimeP4),data = PY4_Jday)### final

***###___GAM______FOR GAM***

summary(YP4gam1)

***#___GAM______GAM_check residuals***

gam.check(YP4gam1)

***#___GAM______GAM_check effect plot***

par(mfrow=c(1,2))

plot(YP4gam2, scheme=1)

par(mfrow=c(1,1))

plot(YP4gam2, scheme=1)

***###___GAM______GAM check response plots***

plot(YP4gam1, all.terms = T, shift = -0.598156 , ylab= "Mean Day Water Tempearture (C)",

xlab = "Time (days)", ylim=c(min(-1.5),max(3.2)),main="gam1 YELCHO P4 /n variability pattern within the period", scheme = 1, pages=1)

points(PY4_Jday $decitimeP4, PY4_Jday $mean_TEMp4, pch=16, cex=0.6)

***###___GAM______GAM_check for autocorrelation***

par(mfrow=c(1,2))

acf(residuals(YP4gam1))

pacf(residuals(YP4gam1))

***###___GAM______helps to find ARMA structure***

eacf(residuals(YP4gam1))

***###::::GAMM::::::::::Autoregression structure GAM1***

YP4_AR1 <- (gamm(mean_TEMp4 ~ s(decitimeP4), data = PY4_Jday, correlation = corARMA(p=1)))

##anova(YP4gamm1$lme,YP4_AR1$lme,YP4_AR1MA1$lme,YP4_AR2MA1$lme)

plot(YP4_AR1$gam,all.terms = T, shift = -0.60589, ylab= "Mean Day Water Tempearture (C)", xlab = "Time (days)", ylim=c(min(-1.5),max(3.2)),main="gam1AR1 YELCHOP4 /n seasonality within a year", scheme = 1, pages=1)

points(PY4_Jday $decitimeP4, PY4_Jday $mean_TEMp4, pch=16, cex=0.6)

***###FOR GAMM_results summary check autocorrelation***

summary(YP4_AR1$gam)

layout(matrix(1:1, ncol = 1))

plot(YP1_AR1MA1$gam, scale = 0)

layout(1)

RESS<-resid(YP4_AR1$lme, type = "normalized")

par(mfrow=c(1,2))

acf(RESS, lag.max = 300, main = "ACF - ARMA errors/ YP4_AR1")

pacf(RESS, lag.max = 300, main = "pACF- ARMA errors/ YP4_AR1")

eacf(RESS)

acf(RESS, plot=FALSE)$acf[2]

**PY1_ Method of finite differences (Curtis and Simpson, 2014: Monteith et al., 2014)**

require(nlme)

require(mgcv)

require(TSA)

require(lubridate)

YP1gam2<-gam(mean_TEMP ~ s(decitime, k=58),data = PY1_Jday)

***##################################################################***

***## Functions for derivatives of GAM(M) models from Curtis and Simpson, 2014##***

***##################################################################***

Deriv <- function(mod, n = 400, eps = 1e-3, newdata, term) {

if(inherits(mod, "gamm"))

mod <- mod$gam

m.terms <- attr(terms(mod), "term.labels")

if(missing(newdata)) {

newD <- sapply(model.frame(mod)[, m.terms, drop = FALSE],

function(x) seq(min(x), max(x), length = n))

names(newD) <- m.terms

} else {

newD <- newdata

}

newDF <- data.frame(newD) ## needs to be a data frame for predict

X0 <- predict(mod, newDF, type = "lpmatrix")

newDF <- newDF + eps

X1 <- predict(mod, newDF, type = "lpmatrix")

Xp <- (X1 - X0) / eps

Xp.r <- NROW(Xp)

Xp.c <- NCOL(Xp)

## dims of bs

bs.dims <- sapply(mod$smooth, "[[", "bs.dim") - 1

## number of smooth terms

t.labs <- attr(mod$terms, "term.labels")

## match the term with the the terms in the model

if(!missing(term)) {

want <- grep(term, t.labs)

if(!identical(length(want), length(term)))

stop("One or more 'term's not found in model!")

t.labs <- t.labs[want]

}

nt <- length(t.labs)

## list to hold the derivatives

lD <- vector(mode = "list", length = nt)

names(lD) <- t.labs

for(i in seq_len(nt)) {

Xi <- Xp * 0

want <- grep(t.labs[i], colnames(X1))

Xi[, want] <- Xp[, want]

df <- Xi %*% coef(mod)

df.sd <- rowSums(Xi %*% mod$Vp * Xi)^.5

lD[[i]] <- list(deriv = df, se.deriv = df.sd)

}

class(lD) <- "Deriv"

lD$gamModel <- mod

lD$eps <- eps

lD$eval <- newD - eps

lD ##return

}

confint.Deriv <- function(object, term, alpha = 0.05, ...) {

l <- length(object) - 3

term.labs <- names(object[seq_len(l)])

if(missing(term)) {

term <- term.labs

} else { ## how many attempts to get this right!?!?

##term <- match(term, term.labs)

##term <- term[match(term, term.labs)]

term <- term.labs[match(term, term.labs)]

}

if(any(miss <- is.na(term)))

stop(paste("'term'", term[miss], "not a valid model term."))

res <- vector(mode = "list", length = length(term))

names(res) <- term

residual.df <- df.residual(object$gamModel)

tVal <- qt(1 - (alpha/2), residual.df)

##for(i in term.labs[term]) {

for(i in term) {

upr <- object[[i]]$deriv + tVal * object[[i]]$se.deriv

lwr <- object[[i]]$deriv - tVal * object[[i]]$se.deriv

res[[i]] <- list(upper = drop(upr), lower = drop(lwr))

}

res$alpha = alpha

res

}

signifD <- function(x, d, upper, lower, eval = 0) {

miss <- upper > eval & lower < eval

incr <- decr <- x

want <- d > eval

incr[!want | miss] <- NA

want <- d < eval

decr[!want | miss] <- NA

list(incr = incr, decr = decr)

}

plot.Deriv <- function(x, alpha = 0.05, polygon = TRUE,

sizer = FALSE, term,

eval = 0, lwd = 3,

col = "lightgrey", border = col,

ylab, xlab, main, ...) {

l <- length(x) - 3

## get terms and check specified (if any) are in model

term.labs <- names(x[seq_len(l)])

if(missing(term)) {

term <- term.labs

} else {

term <- term.labs[match(term, term.labs)]

}

if(any(miss <- is.na(term)))

stop(paste("'term'", term[miss], "not a valid model term."))

if(all(miss))

stop("All terms in 'term' not found in model.")

l <- sum(!miss)

nplt <- n2mfrow(l)

tVal <- qt(1 - (alpha/2), df.residual(x$gamModel))

if(missing(ylab))

ylab <- expression(italic(hat(f)*"'"*(x)))

if(missing(xlab)) {

xlab <- attr(terms(x$gamModel), "term.labels")

names(xlab) <- xlab

}

if (missing(main)) {

main <- term

names(main) <- term

}

## compute confidence interval

CI <- confint(x, term = term)

## plots

layout(matrix(seq_len(l), nrow = nplt[1], ncol = nplt[2]))

for(i in term) {

upr <- CI[[i]]$upper

lwr <- CI[[i]]$lower

ylim <- range(upr, lwr)

plot(x$eval[,i], x[[i]]$deriv, type = "n",

ylim = ylim, ylab = ylab, xlab = xlab[i], main = main[i], ...)

if(isTRUE(polygon)) {

polygon(c(x$eval[,i], rev(x$eval[,i])),

c(upr, rev(lwr)), col = col, border = border)

} else {

lines(x$eval[,i], upr, lty = "dashed")

lines(x$eval[,i], lwr, lty = "dashed")

}

abline(h = 0, ...)

if(isTRUE(sizer)) {

lines(x$eval[,i], x[[i]]$deriv, lwd = 1)

S <- signifD(x[[i]]$deriv, x[[i]]$deriv, upr, lwr,

eval = eval)

lines(x$eval[,i], S$incr, lwd = lwd, col = "blue")

lines(x$eval[,i], S$decr, lwd = lwd, col = "red")

} else {

lines(x$eval[,i], x[[i]]$deriv, lwd = 2)

}

}

layout(1)

invisible(x)

}

***###Derivative code***

YEL1.deriv1=Deriv(YP1gam2, n=404)

plot(YEL1.deriv1, sizer=TRUE, alpha=0.01)##first derivative plot

***###Plot***

plot(mean_TEMP ~ decitime, data = PY1_Jday, type = "p", ylab = "Water Temperature")

pdat <- with(PY1_Jday, data.frame(decitime = seq(min(decitime), max(decitime), length = 404)))

p2 <- predict(YP1gam2, newdata = pdat)

lines(p2 ~ decitime, data = pdat)

CI <- confint(YEL1.deriv1, alpha = 0.01)

S <- signifD(p2, YEL1.deriv1$decitime$deriv, CI$decitime$upper, CI$decitime$lower,

eval = 0)

lines(S$incr ~ decitime, data = pdat, lwd = 3, col = "blue")

lines(S$decr ~ decitime, data = pdat, lwd = 3, col = "red")

***##Export data from method of finite differences to csv file***

blueSIGPLOT<-S <- signifD(p2, YEL1.deriv1$decitime$deriv, CI$decitime$upper, CI$decitime$lower, eval = 0)

write.csv( blueSIGPLOT, file = "blueSIGPLOT.csv")
